# Protocol: investigating the effectiveness and cost benefit of a lifestyle intervention targeting type 2 diabetes in Australia

**DOI:** 10.1186/s12902-019-0396-x

**Published:** 2019-07-15

**Authors:** Linda Cloete, Brett G. Mitchell, Darren Morton

**Affiliations:** 1 Faculty of Arts Nursing and Theology, Avondale College of Higher Education, 185 Fox Valley Road, Wahroonga, Sydney, NSW 2076 Australia; 2 School of Nursing and Midwifery, Faculty of Health and Medicine, University of Newcastle, Q-10 Loop Road & The Boulevarde, Ourimbah, NSW Australia; 3 Faculty of Education and Business, Avondale College of Higher Education, 582 Freemans Drive, Cooranbong, NSW Australia

**Keywords:** Type 2 Diabetes Mellitus, Lifestyle, Diabetes, CHIP, Cost benefit

## Abstract

**Background:**

Type 2 Diabetes Mellitus (T2DM) has become an endemic disease. A number of interrelated factors increase the risk of the onset of T2DM, however much of the pathogenesis of the disease is associated with lifestyle. A number of studies have indicated that adopting positive lifestyle changes can successfully prevent or delay the onset of T2DM in a number of different population groups. The CHIP intervention is a lifestyle program that has been shown in over more than 30 published papers have indicated that the CHIP intervention leads to dramatic improvement in the indicators of T2DM these diseases of lifestyle.

**Methods:**

A randomized control trial will be conducted involving 150 individuals with an established diagnosis of T2DM. All participants will continue to receive usual ongoing diabetes care, however, the intervention group (75 individuals) will in addition participate in a 12-week CHIP lifestyle intervention programme followed by a further 9 months of monthly follow-up appointments. Approval for funding was obtained on 30 June 2017.

**Discussion:**

The outcomes of this study have the potential to inform decisions about patient treatment and potentially provide incentive for the provision of funded lifestyle-based preventive and restorative programs for patients diagnosed with T2DM.

**Trial registration:**

This trial is registered as an initial version with the Australia New Zealand Clinical Trials Registry (http://www.anzctr.org.au/), registration number ACTRN12617001233314. Registered on 23/08/2017. No enrollments in the study to date.

## Background

Type 2 Diabetes Mellitus (T2DM) has become an endemic disease [1]. A number of interrelated factors increase the risk of the onset of T2DM, however much of the pathogenesis of the disease is associated with lifestyle, particularly the lifestyle habits associated with obesity [2].

The relationship between the onset of insulin resistance and the presence of obesity has been recognized for decades and is present in all ethnic groups and across the full spectrum of age, gender and body weight [3–5]. Both total and central (intra-abdominal) adiposity are strongly linked to insulin resistance as well as to other metabolic variables including increased plasma glucose, insulin, blood pressure, total cholesterol, low density lipoprotein (LDL) cholesterol and triglyceride concentrations and decreased plasma high-density lipoprotein (HDL) cholesterol concentration [6, 7].

Data from the DECODE study [8] indicated that myocardial infarction (MI), stroke or non-ischaemic cardiovascular disease cause 80% of deaths among patients with T2DM and over 60% of people having T2DM will develop cardiovascular disease. Indeed, T2DM and associated insulin resistance have been recognized as factors that independently increase the risk of cardiovascular morbidity and mortality. They also act synergistically with other major risk factors for cardiovascular disease such as smoking, hypertension and dyslipidaemia to increase cardiovascular disease risk [9].

A number of studies have indicated that adopting positive lifestyle changes can successfully prevent or delay the onset of T2DM in a number of different population groups [10–13]. Lifestyle changes recommended in these studies relate to those associated with reducing obesity such as increasing physical activity levels and consuming a low energy-density diet [13]. The eating pattern recommended in these studies was largely comprised of plant based foods that are low fat and cholesterol but high in fibre, which is in contrast to the traditional Western diet.

The Complete Health Improvement Program (CHIP) is a lifestyle intervention that was primarily designed to reverse coronary artery disease (CAD). Since its inception in 1988, researchers have become increasingly aware that the intervention was also effective in improving clinical outcomes for diseases like hypertension and T2DM. More than 30 published papers have indicated that the CHIP intervention leads to dramatic improvement in the indicators of these diseases of lifestyle. [14–17].

With no known medical cure for T2DM as well as the current and predicted increasing prevalence of the disease, it is important that the benefits of improved lifestyle in the population with diabetes continue to be explored. Increasing healthcare costs associated with chronic diseases and their complications make it necessary to employ programs that are effective and demonstrate a cost benefit.

## Methods/design

### Aims and research questions

There are two aims of this study:

1. To assess the effectiveness of the CHIP intervention in reversing abnormal fasting blood sugar levels in people with T2DM.
2. To determine the cost benefit of the CHIP intervention for persons with a diagnosis of T2DM.

The study will address three main questions;

In a diabetic population within a general practice group on the Central Coast of New South Wales, Australia:

1. When compared to a control group receiving routine diabetic care, what changes occur in fasting blood sugar levels (FBSL), HBA1c, fasting lipogram with triglycerides (FLT), blood pressure (BP), abdominal girth, body mass index (BMI) and medication use among  individuals participating in the CHIP intervention delivered over a period of 12 weeks with additional monthly follow up for another 9 months?
2. Over a 12-month period, what levels of compliance with the intervention are observed in participants?
3. When compared to routine diabetic care, what is the cost benefit (over a 12-month period) of implementing a CHIP intervention delivered over a period of 12 weeks with an additional follow up for another 9 months?

### Study design

A parallel randomized control study design will be used. A cohort of 150 patients will be randomized into either an intervention or control group. There will be 75 participants in each of the two groups. For the purpose of this study Group 1 will be the intervention group and Group 2 the control group.

### Study setting

The CHIP intervention will be conducted by CHIP trained volunteers in selected community halls on the Central Coast of NSW. A demographic and lifestyle survey will be administered at the venue the intervention is conducted. Anthropometric measurements and blood collection will occur at the venue at which the CHIP intervention is being conducted unless the facility is not suited for medical data collection in which case it will be collected at selected general practices.

### Study participants

- Patients, who have a diagnosis of T2DM will be recruited from general practice medical centers (where practitioners have agreed to refer) on the Central Coast of NSW, Australia. Recruited patients will be invited to participate in the study. Enrollment of consenting participants will continue until the required sample size is met. The source population will be monitored at each participating General medical practice in order to ascertain what percentage of diabetes patients would be suitable to join the programme. Potential participants will be excluded based on the criteria below.

### Exclusion criteria

Persons will be ineligible for the study if:

- they are aged less than 18 years
- the consulting medical practitioner considers that an alteration in nutrient or calorie consumption or that participating in regular moderate exercise could result in an increased health risk. This may include but may not be limited to:
  i. persons having a history of uncontrolled hypertension with consistent resting blood pressure readings greater than 180 mmHg systolic and or greater than 110 mmHg diastolic;
  ii. persons having a BMI lower than 21 kg/m^2^, and making use of weight-loss medication;
  iii. persons experiencing recurrent chest pain or unstable angina;
  iv. pregnant or lactating females, or females planning to become pregnant in the next year;
  v. persons who have undergone angioplasty or experienced a myocardial infarction or cardiac surgery within the previous three months;
- they have any condition that precludes them from increasing their level of physical activity;
- they have any physical condition or language barrier that would preclude them from understanding or safely adopting the lifestyle changes recommended by the CHIP intervention.

### Allocation of participants to a study group

Invitees who are willing to participate will be recorded on a list until such time as there are between 30 and 40 individuals listed. At this point, the individual participants will be randomized into either Group 1 or Group 2.

Once allocated, participants in Group 1, will receive the CHIP intervention over a period of 12 weeks. At the end of the 12 weeks period, Group 1 will receive monthly follow-up for a further 9 month period. This process will be repeated, until the full number of participants have been collected and assigned.

**Fig. 1:**
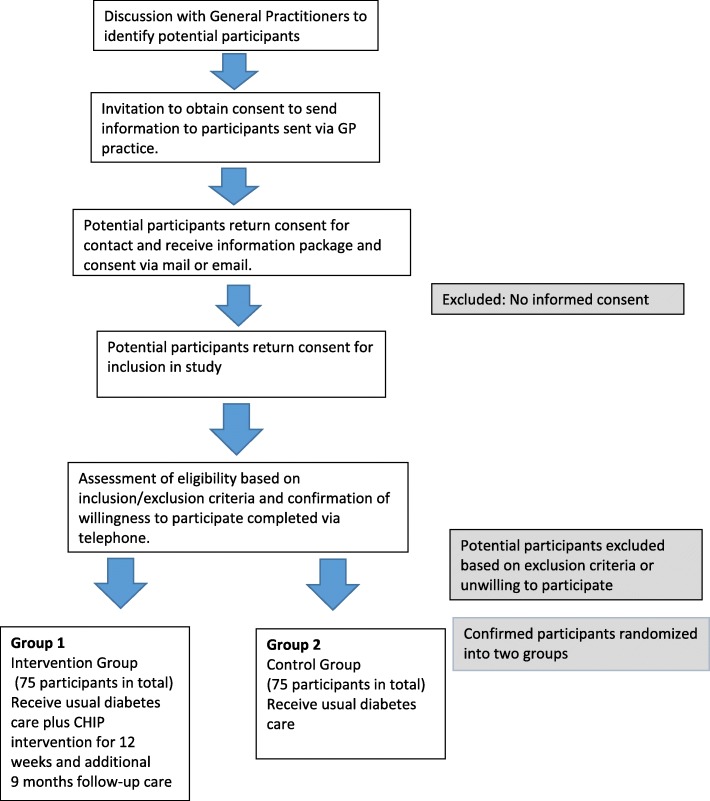
An overview of the study design and allocation of participants

An overview of the study design and allocation of participants is illustrated in Fig. 1.

### Randomisation

The randomisation of participants will be performed by a person independent of the research team. The process of randomisation will occur with the aid of a computer. No blinding will be applied.

### Interventions

#### Group 1- intervention group

All participants in Group 1 will participate in the CHIP intervention for 12 weeks. The CHIP intervention consists of 18 education sessions, each of 90 min duration. Approximately half of each session is devoted to viewing pre-recorded visual material delivered by lifestyle medicine professionals, while interactive group discussions and activities, including food preparation and exercise demonstrations delivered by CHIP trained volunteers, make up the remaining time. An outline of the topics covered over the 18 sessions can be viewed in Table 1.

**Table 1:** CHIP sessions as per topic

Session number	Topic
1	The rise of chronic disease
2	Lifestyle as medicine
3	The common denominator of chronic disease
4	The optimal lifestyle
5	Eat more, weigh less
6	Fibre, your new best friend
7	Disarming Diabetes
8	The heart of the matter – heart healthy
9	Controlling blood pressure and discovering protein
10	Bone health essentials
11	Cancer prevention
12	Understanding your results and taking action
13	Become what you believe. Your DNA is not your destiny
14	Practicing forgiveness
15	Re-engineering your environment
16	Stress relieving strategies
17	Fix how you feel
18	From surviving to thriving

Sessions 1 to 11 provides information on the aetiology, risk factors and pathological mechanism responsible for lifestyle related chronic disease. Emphasis is placed on the links between obesity, diabetes, hypertension, hyperlipidaemia and cardiovascular disease. Participants are provided with information relating to how lifestyle changes, particularly in diet and levels of physical activity, can positively alter the risk factors for as well as the course of chronic diseases. Specifically, the adoption of a plant-based, restricted sugar and salt diet is encouraged as well as daily physical activity involving a combination of aerobic and strengthening exercise most days of the week. Sessions 12 to 18 provide information related to other important lifestyle behaviors like substance use, rest, and sleep and stress management. Participants are provided with strategies that will assist them in assuming a position of control of their health, to overcome barriers, to manage and maintain behavior change and to cope with environmental pressures and stressors. At the end of the 12-week program, participants will be contacted for the purpose of assessing compliance and providing motivation and support on a monthly basis for an additional 9 months. Participants who receive less than 80% (4 sessions) of the program content will be regarded as having dropped out from the intervention and therefore will be excluded from the study. Self reported reasons for dropout will be noted.

#### Group 2 – control group

The control group will continue to receive their usual diabetes care and not receive any intervention or details about the CHIP intervention.

### Data collection

Data collection consists of a standard participant survey (including questions on food frequency, daily levels of occupational and recreational activity, smoking, hours of sleep, medical conditions and medication use), anthropometric data, blood tests, an inventory of activity, the completion of validated health, stress and diabetes quality of life surveys and cost. The costs will include cost associated with the program, monitoring and ongoing medical care. Initial data collection will occur prior to commencement of the CHIP intervention. Repeat data collection time points for both groups will be at the end of the 12-week intervention and again at 12 months from the initial commencement date.

### Sample size estimate

The total sample required for this study is 150, with an equal number of participants in each arm of the study. The sample size was calculated on the following assumptions: a reduction in 3.5% of fasting glucose in the intervention group, an equal allocation of participants to each group as well as a 10% drop out rate. A standard deviation of 20 was used. This study is sufficiently powered d > 80% to detect a 3.5% reduction in fasting glucose using significance of 0.05 (95% confidence interval).

### Statistical analysis

All data collected will be coded and entered into IBM® SPSS® version 22 for Windows® (SPSS Inc., 2013) before analysis.

#### Analysis for research question 1 and 2

Descriptive statistics will be used to analyze demographic data, anthropometric measurement data, blood results and activity levels on commencement of the program and also at the described re-evaluation intervals. Descriptive statistics will include frequencies, percentage distribution, means, and medians as well as standard deviation from the mean.

The Shapiro-Wilk test will be used to determine normal distribution to decide on whether to use parametric or non-parametric testing for correlations and variance analysis. A paired sample t-test (dependent t-test) will be used to compare pre intervention data with post intervention (12 weeks) and pre intervention data with post intervention (12 months) data. An independent t-test will be used to compare data between intervention and control groups. Pearson’s correlation will be used to explore relationships between continuous variables. The dietary inventory, health survey, and quality of life survey will be independently scored and the results between groups compared using linear regression. The level of significance is set at *p* < 0.05.

#### Analysis for research question 3

To determine the cost benefit of the intervention, a cost-benefit analysis will be undertaken. The cost benefit analysis will involve assessment of beneficial effects of the intervention. The analysis will include benefits and costs attributed to the project and indirect costs. Therefore, where B represents all the benefits and C represents all the costs, the project will be considered beneficial if B-C > 0. A cost benefit ratio will also be calculated. Costs included in the analysis will include opportunity costs. Costs from published literature and from government agencies will be used wherever possible, for example, the Medical Benefits Scheme and Pharmaceutical Benefits Scheme.

## Dissemination of results

Results of this study will be disseminated to participants through the means of a newsletter. It is also the authors intention to communicate results through conference proceedings and journal publication. This protocol as well as further results are required to be presented at research forums held by Avondale College of Higher Education.

## Discussion

This study addresses a gap in knowledge, in that while there have been a number of studies exploring changes in diet and or exercise and effect on T2DM, no specific randomized controlled study investigating the effectiveness of a holistic health programme in targeting T2DM has been undertaken. Further, while a number of  studies using the CHIP lifestyle programme have indicated significant improvements in blood glucose and lipid profiles, the proposed study investigates the cost benefit of such a programme, thus informing decisions about appropriate allocation of scare health resources.

### Ethical considerations

Ethics has been approved by Avondale Human Research Ethics Committee. Approval identification number 2017:02. The specific principles relevant to this study are research merit and integrity, justice, beneficence and respect (The National Health and Medical Research Council et al., 2007 Updated 2015).

This study has been designed to be low risk, and is likely to provide valuable insight into lifestyle management of T2DM. Patients approached for participation will be offered equal opportunity to participate in the study. No potential participants will be coerced and no monetary or other rewards offered other than potential health benefits achieved. The outcomes of the study will be made known to all participants. Other than laboratory testing requiring the drawing of blood samples no harm to participants is anticipated. Informed consent will be obtained from all participants. Participants may elect to opt out of answering questions, participating in surveys and are free to exit the study at any time without any consequence to their medical management. All data will be collected in a de-identified format using coding to match data. Once coding has been completed, the original coding descriptors will be destroyed. Following the data analysis process, any information that allows the data to be identified in any way will be permanently destroyed. Data collected will be entered into SPSS and stored on an external hard drive which will be kept under lock and key while not in use for analysis. All original data will be stored in paper format in a locked filing cabinet in the researcher’s office on Avondale College of Higher Education’s Sydney campus until the completion of data collection and analysis. Thereafter the data will be permanently destroyed. The results of this study will be published in a format that will protect all participant’s identity. No individual results will be published. Participants will not be required to personally fund any part of the study or laboratory tests required for data collection.

### Medical management of participants

Participants will be referred to their General Practitioner for any medically related symptoms or medication changes that may be required due to alterations in blood sugar or blood pressure measurements.

### Limitations

Several factors may influence the results obtained in this study. Firstly, compliance with the intervention is paramount to the success of lifestyle modification programmes, but it is also difficult to measure actual compliance reliably. Some data collection, namely dietary analysis, exercise compliance and medical costs will rely on patient integrity and accurate reporting which may not necessarily be reliable.

A potential confounder is that of the Hawthorne effect in that the participants’ behaviour may be affected by the fact that data is collected at baseline and again at future intervals.

The outcomes of this study have the potential to inform decisions about patient treatment and potentially provide incentive for the provision of funded lifestyle- based preventive and restorative programs for patients diagnosed with T2DM.

### Study status

At the time of submission for publication the researcher is about to begin the process of participant recruitment.

## Conclusion

The outcomes of this study have the potential to inform decisions about patient treatment and potentially provide incentive for the provision of funded lifestyle based preventive and restorative programs for patients diagnosed with T2DM.

## Data Availability

Not applicable.

## Abbreviations

BGL: Blood glucose level
BMI: Body mass index
CAD: Coronary artery disease
CHIP: Complete Health Improvement Programme
LDL: Low-density lipoprotein cholesterol
NSW: New South Wales
T2DM: Type 2 Diabetes Mellitus
